# Safety and efficacy of human juvenile chondrocyte-derived cell sheets for osteochondral defect treatment

**DOI:** 10.1038/s41536-021-00173-9

**Published:** 2021-10-15

**Authors:** Makoto Kondo, Sumako Kameishi, Kyungsook Kim, Nicolas F. Metzler, Travis G. Maak, Douglas T. Hutchinson, Angela A. Wang, Miki Maehara, Masato Sato, David W. Grainger, Teruo Okano

**Affiliations:** 1 Cell Sheet Tissue Engineering Center (CSTEC), Department of Pharmaceutics and Pharmaceutical Chemistry, Health Sciences, University of Utah, 30 South 2000 East, Salt Lake City, UT 84112 USA; 2 Department of Biomedical Engineering, University of Utah, 36 S. Wasatch Drive SMBB 3100, Salt Lake City, UT 84112 USA; 3 Department of Orthopaedic Surgery, University of Utah Orthopaedic Center, University of Utah, 590 Wakara Way, Salt Lake City, UT 84108 USA; 4 Pediatric Orthopaedics Surgery, Primary Children’s Hospital Orthopedics, 100 North Mario Capecchi Dr. Suite 4550, Salt Lake City, UT 84113 USA; 5 Department of Orthopaedic Surgery, Surgical Science, Tokai University School of Medicine, 143 Shimokasuya, Isehara, Kanagawa 259-1193 Japan; 6 Institute of Advanced Biomedical Engineering and Science, Tokyo Women’s Medical University, TWIns, 8-1 Kawada-cho, Shinjuku-ku, Tokyo 162-8666 Japan

**Keywords:** Trauma, Cartilage, Regeneration, Preclinical research, Tissue engineering

## Abstract

Knee cartilage does not regenerate spontaneously after injury, and a gold standard regenerative treatment algorithm has not been established. This study demonstrates preclinical safety and efficacy of scaffold-free, human juvenile cartilage-derived-chondrocyte (JCC) sheets produced from routine surgical discards using thermo-responsive cultureware. JCCs exhibit stable and high growth potential in vitro over passage 10, supporting possibilities for scale-up to mass production for commercialization. JCC sheets contain highly viable, densely packed cells, show no anchorage-independent cell growth, express mesenchymal surface markers, and lack MHC II expression. In nude rat focal osteochondral defect models, stable neocartilage formation was observed at 4 weeks by JCC sheet transplantation without abnormal tissue growth over 24 weeks in contrast to the nontreatment group showing no spontaneous cartilage repair. Regenerated cartilage was safranin-O positive, contained type II collagen, aggrecan, and human vimentin, and lacked type I collagen, indicating that the hyaline-like neocartilage formed originates from transplanted JCC sheets rather than host-derived cells. This study demonstrates the safety of JCC sheets and stable hyaline cartilage formation with engineered JCC sheets utilizing a sustainable tissue supply. Cost-benefit and scaling issues for sheet fabrication and use support feasibility of this JCC sheet strategy in clinical cartilage repair.

## Introduction

Articular cartilage plays an essential role in reducing friction in joint motion and mitigating joint load stress. In contrast to bone, the post-traumatic repair capacity of cartilage is limited^1^. As such, focal cartilage defects have been identified as a potent risk factor for early osteoarthritic disease^2,3^, and restoring cartilage integrity is considered to be a key means of preventing or delaying the reduction in patient quality of life associated with osteoarthritis development^4,5^. Autologous and allogeneic osteochondral grafts have been applied as mosaicplasty, bulk grafts, and particulated cartilage^6^ to replace damaged cartilage. However, allogeneic grafts have limited supply, treatable lesion location, and area restricts graft options, and the limited duration of bulk osteochondral allograft (OCA) transplantation viability makes the timing of surgical treatment difficult^7^. Finally, in many cases, fibrocartilage is often reported following chondral repair techniques.

After a milestone report of in vitro cultured autologous chondrocyte implantation^8^, various tissue engineering methods using chondrocytes and mesenchymal stem/stromal cells (MSCs), with or without accompanying biomaterials, have been developed with a myriad of clinical studies ongoing^9–12^. Juvenile cartilage is noted as a desirable source for neocartilage regeneration due to its immune tolerance^13^, proliferative capacity, and maintenance of characteristic matrix proteins in vitro compared to adult cartilage-derived chondrocytes^14,15^. Allogeneic sourcing reduces chondral defect treatment to a single-stage surgical procedure^16,17^. However, the adhesion of chondral grafts to cartilage and bone surfaces is difficult and graft failure is reported^18^.

Cell sheet technology using poly(*N*-isopropylacrylamide)-grafted temperature-responsive cultureware allows reproducibly consistent creation of sheets via detachment of confluently cultured cells^19^. Harvested sheets readily adhere on tissue lesions with endogenous extracellular matrix (ECM) and surface proteins eliminating secondary adhesion techniques, such as suture or fibrin glue. Cell sheets are applied to treat multiple diseases in patients using various cell types^20–25^. The safety and clinical efficacy of autologous chondrocyte sheet transplantation was demonstrated in a small cohort study with concomitant anterior cruciate ligament reconstruction or high tibial osteotomy (HTO) and microfracture^26^. Recently, surgically discarded juvenile tissue obtained from routine polydactyly resection is reported as a source of allogeneic human chondrocytes to form hyaline cartilage in implanted subcutaneous space of immunodeficient mouse^27,28^. Polydactyly-derived chondrocyte sheets were compared to adult chondrocyte sheets fabricated from adult tissue discards obtained from patients undergoing total knee arthroplasty and shown to exhibit higher secreted TGFβ1 concentrations, with practical advantages useful to future cartilage repair^29^.

The current study reports the preclinical safety and efficacy of polydactyly-sourced juvenile cartilage-derived chondrocyte (JCC) sheets in vitro and in vivo using a rat focal osteochondral defect model. Properties of these JCC sheets in sheet production and rodent cartilage repair suggest feasibility for possible future clinical translation of this therapy.

## Results

### In vitro characterization of juvenile cartilage-derived chondrocytes

**Fig. 1:**
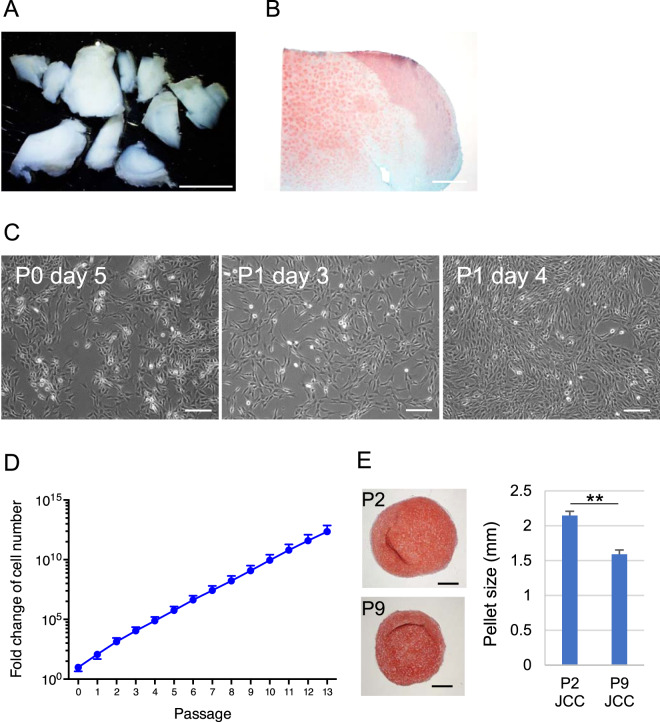
Isolation of chondrocytes from juvenile cartilage surgical discards and in vitro expansion of juvenile chondrocytes. **A** Juvenile donor-derived cartilage tissues under stereomicroscope. Scale bar: 5 mm. **B** Safranin-O staining of cartilage tissue. Scale bar: 500 μm. **C** Phase-contrast images of cultured chondrocytes. Scale bars: 200 μm. **D** Average fold-change of in vitro cell expansion. Data shown as mean and SD (*n* = 13 individual donors). **E** In vitro differentiated JCC pellets. Photos show Safranin-O staining of P2 JCCs (top) and P9 JCCs (bottom). Bars: 500 µm. Right graph shows pellet size diameter measurements (*n* = 2). Error bars indicate SD. **p < 0.01 by Student’s *t* test.

To demonstrate the safety of juvenile cartilage-derived-chondrocyte (JCC) sheets, juvenile cartilage tissue, and JCCs in culture were characterized. Surgically discarded polydactyly cartilage samples from 12 juvenile human donors (Table 1) were harvested (Fig. 1A) and confirmed to contain hyaline cartilage using safranin-O staining (Fig. 1B). The morphology of isolated chondrocytes was observed after initial attachment and during subsequent culture. The chondrocytes showed stellate shapes after surface attachment, then spread after one passage (Fig. 1C). These cells exhibit a constant growth rate for over 10 passages (Fig. 1D). Harvested JCCs at the end of P2 and P9 both formed fully differentiated hyaline cartilage pellets after 3-week differentiation culture although the size of P9 pellets were slightly smaller compared to P2 pellets (Fig. 1E), suggesting decreased growth potential, but maintained differentiation potential at P9.

**Table 1:** Juvenile chondrocyte donor sample information.

Donor ID	Age (months)	Sex	Ethnicity	Anatomical site	Viability at end of P1	Cell sheet formation	CS detachment test	Total cell number of CS	Cell viability of CS	Serial passage culture	CS Histology	Transformation assay	Surface marker	In vivo experiment
1	16	M	Cauc.	finger	97.2%	✔	✔	✔	✔	✔	✔	✔	✔	
2	22	M	Cauc.	finger	97.2%	✔	✔	✔	✔	✔	✔	✔	✔	
3	24	F	Cauc.	finger	97.1%	✔	✔	✔	✔	✔	✔	✔	✔	
4	48	M	Cauc.	finger	98.8%	✔	✔	✔	✔	✔	✔	✔	✔	✔
5	19	M	Cauc.	finger	99.5%	✔	✔	✔	✔	✔	✔			✔
6	13	F	Cauc.	finger	99.1%	✔	✔	✔	✔	✔	✔			✔
7	14	F	Cauc.	finger	100.0%	✔	✔	✔	✔	✔	✔		✔	✔
8(f)	30	M	Cauc.	finger	99.4%	✔	✔	✔	✔	✔	✔			✔
8(t)	30	M	Cauc.	toe	99.4%	✔	✔	✔	✔	✔	✔			✔
9	9	M	Cauc.	finger	99.2%	✔	✔	✔	✔	✔	✔			✔
10	72	F	Cauc.	finger	99.8%	✔	✔	✔	✔	✔	✔		✔	✔
11	24	M	Cauc.	toe	99.8%	✔	✔	✔	✔	✔				
12	7	M	Cauc.	finger	98.2%	✔	✔	✔	✔	✔

### In vitro characterization of juvenile cartilage-derived-chondrocyte sheets

**Fig. 2:**
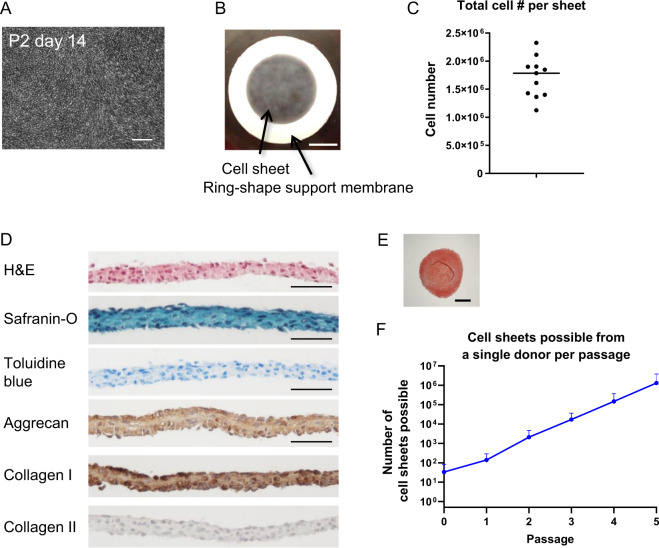
Characterization of engineered JCC sheet. **A** A phase-contrast image of confluent chondrocytes at day 14 of passage 2. Scale bar: 200 µm. **B** Macroscopic image of engineered JCC sheet. Scale bar: 5 mm. **C** Total cell number in one cell sheet (*n* = 13 individual donors). **D** Hematoxylin and eosin staining, Safranin-O staining, Toluidine blue staining, aggrecan, type I collagen, and type II collagen immunohistochemistry of JCC sheets. Bars: 50 µm. **E** Safranin-O staining of an in vitro differentiated pellet from P2 JCC sheet. Bar: 500 µm. **F** Theoretical cell sheet numbers possibly prepared from cultured JCCs at each passage. Data shown as mean and SD (*n* = 11 individual donors).

P2 JCCs cultured on temperature-responsive cell culture insert for 2 weeks were confluent (Fig. 2A) and able to be harvested as cell sheets (Fig. 2B). JCC sheets maintain high cell viability (98.0 ± 1.3%) and rich cell numbers in each sheet construct (1.90 ± 0.48 × 10^6^ cells per sheet) (Fig. 2C). After detachment, JCC sheets undergo a spontaneous, endogenous contraction resulting in a multicell thick sheet structure without folding (Fig. 2D). The cell sheet stains negatively for safranin-O and toluidine blue (only at nuclei), but positively for aggrecan and type I collagen with immunohistochemistry (Fig. 2D). The expression of type II collagen was not evident in the cell sheets. These in vitro characters were maintained in the cell sheets prepared at passage 9 (Supplementary Fig. 2). Importantly, cells isolated from JCC sheets exhibit robust chondrogenic capacity in pellet cultures (Fig. 2E). Theoretical numbers of JCC sheets prepared from a single polydactyly donor at a given passage are shown in Fig. 2F.

### In vitro safety evaluation of juvenile cartilage-derived-chondrocyte sheets

**Fig. 3:**
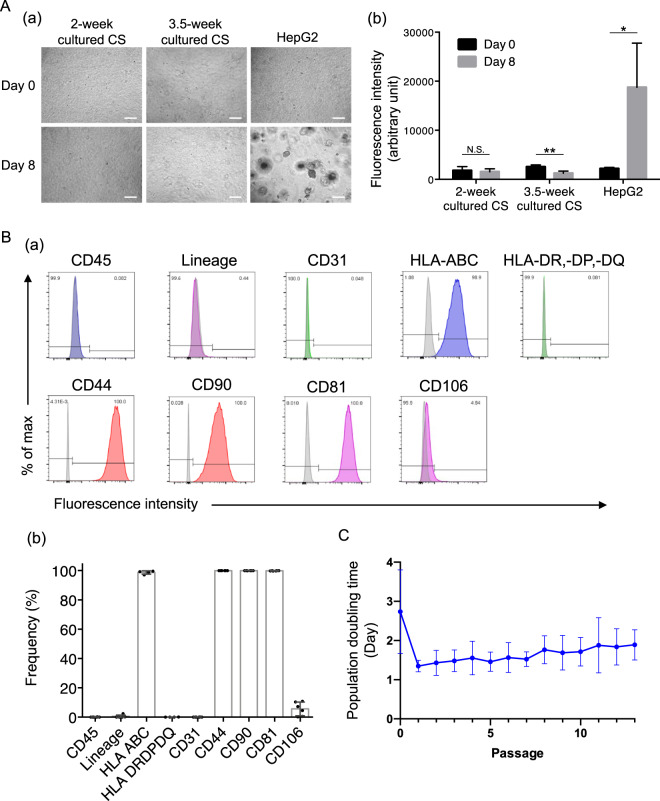
Tumorigenicity assay, population doubling time, and cell surface markers. **A** (a) Microscopic images of in vitro tumorigenicity assay in soft agar culture conditions. Images of the left column show seeded cells from 2-week cultured cell sheet. Images of the middle column show seeded cells from 3.5-week cultured cell sheet. Images of the right column show seeded HepG2 cells as a positive control. The top row shows the image at day 0 and the bottom shows images at day 8 after seeding. Bars: 200 µm. (b) Semi-quantification of cell number by DNA-bound fluorescence in soft agar culture at day 0 and day 8. Data shown as mean and SD (*n* = 4 individual donors). **p < 0.01, *p < 0.05, N.S. (non-significant) by Student’ *t* test. **B** Flow cytometry analysis for cell purity and surface marker characterization. (a) Representative histograms for CD45, lineage cocktail (mixture of CD3, CD14, CD16, CD19, CD20, CD56), CD31, HLA-ABC, and HLA-DR, -DP, -DQ, CD44, CD90, CD81, and CD106. Column colors represent fluorophores (blue: Pacific blue, green: FITC or Alx488, red: PE, magenta: APC or Alx647) (b) Average percentages for CD45, lineage cocktail (mixture of CD3, CD14, CD16, CD19, CD20, CD56), CD31, HLA-ABC and HLA-DR, -DP, -DQ, CD44, CD90, CD81, and CD106. *n* = 4–6 individual donors. **C** Population doubling time in the extended subculture in chondrocyte culture medium up to P13. Data shown as mean and SD (*n* = 13 individual donors).

JCC isolated from sheets prepared from four individual donors showed no anchorage-independent colony growth in 10,000 seeded cells (<0.01%) (Fig. 3A(a)). Cells from both regular duration (2-week) cultured cell sheets and extended (3.5-week) cultured JCC sheets showed no increased signal after 8-day soft agar culture, suggesting no anchorage-independent cell growth, whereas HepG2 cells (positive control) showed significant cell growth at 8 days after seeding (Fig. 3A(b)). Cell surface markers for leukocytes (CD45 and lineage cocktail) and vascular endothelial cells (CD31) were negligible (Fig. 3B), indicating that the isolation and culture processes are free of contaminants. MHC-I molecules, HLA-ABC, were expressed 100% whereas MHC-II molecules, HLA-DR, -DP, -DQ, were not detected in the cells isolated from JCC sheets (Fig. 3B), suggesting low allogeneic immunogenicity in long-term graft retention. Expression of mesenchymal cell markers, CD44, CD90, and CD81, were 100%, suggesting pure populations of cultured chondrocytes as previously reported^29^. By contrast, variability of CD106 expression among donors was observed (5.6 ± 4.6%), indicating CD106 cannot be used as a purity marker. To establish phenotypic stability, JCC were serially passaged and morphology and population doubling times at each passage were evaluated. We observed constant cell growth in serial passage culture up to P13 (Fig. 3C), which suggests that engineered juvenile chondrocyte sheets are unlikely to undergo a malignant transformation during a production process.

### In vivo safety and efficacy evaluation

**Fig. 4:**
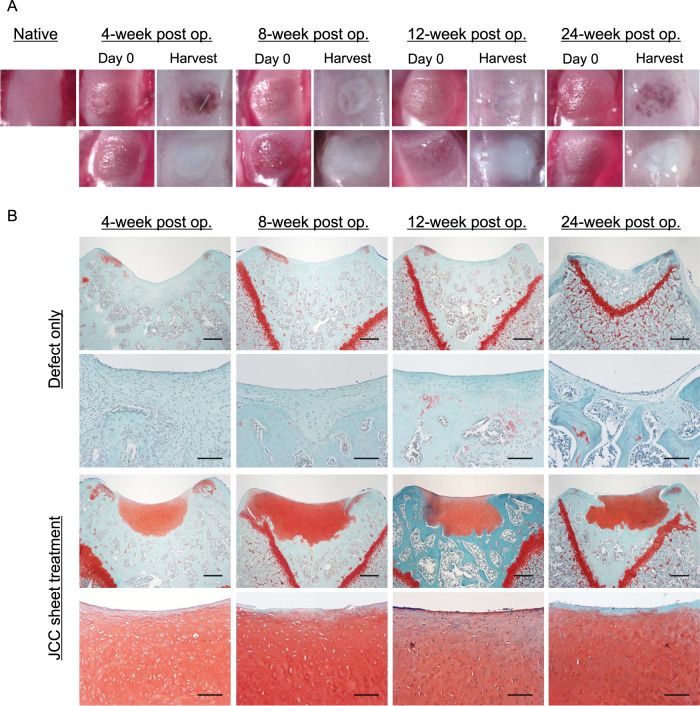
Middle- and long-term in vivo efficacy of focal osteochondral defect treatment in nude rats. **A** Macroscopic images of surgically created focal defects (left images of each group) and 4, 8, 12, and 24 weeks after treatments (right images of each time point). The top row shows native and defect only group. The bottom row shows defect and cell sheet group. **B** Safranin-O staining of each condition. Representative images of samples at 4 weeks (*n* = 14), 8 weeks (*n* = 3), 12 weeks (*n* = 3), and 24 weeks (*n* = 3) are shown. Bars: Top and third rows: 500 µm, second and bottom rows: 100 µm.

To demonstrate both safety and efficacy in vivo, JCC sheets were transplanted at the time of surgical focal osteochondral defect creation in rats. The two experimental groups, (JCC sheet treatment group and defect-only negative control group) were observed under stereomicroscopy, and histologically examined and compared post-transplantation. Preliminary JCC sheet transplantation studies using immunocompetent Sprague Dawley (SD) rats failed to regenerate cartilage (Supplementary Fig. 3). Therefore, an immunocompromised athymic rat model was selected to evaluate sheet-induced neocartilage formation. Seven-week-old nude rats received focal osteochondral defects in the trochlear groove (2-mm diameter, 200–350 µm depth. Depressed knee surface and fibrotic pannus indicative of failure to spontaneously regenerate cartilage tissue were observed in the defect-only group at all-time points (4, 8, 12, and 24 weeks) (Fig. 4B). This is consistent with previously reported critical size defects (>1.4 mm diameter) in rat knee cartilage^30^. Contrary to the defect-only group, the treatment group showed regenerated, white cartilage in defect areas at all time points (Fig. 4A). Moreover, histological examination revealed the defect-only group to be safranin-O negative, whereas the treatment group exhibited thick, safranin-O positive, hyaline neocartilage at all-time points (4, 8, 12, and 24 weeks) (Fig. 4B). Importantly, the interface between the regenerated cartilage and host tissue is stably integrated (Fig. 4B). In addition, samples at all time points exhibited lacuna formation, suggesting mature cartilage formation in defect areas by 4 weeks post-transplantation, and maintenance of native tissue architecture. Interestingly, the size of lacuna was smaller than host native cartilage (Supplementary Fig.. 4), indicating that tissue is not the residue of the original host cartilage. Moreover, while regenerated cartilage was substantially thicker than rat native cartilage, no tumorigenic tissue formation was observed in all rats, suggesting the in vivo safety of transplanted JCC sheets throughout 24 weeks of study.

**Fig. 5:**
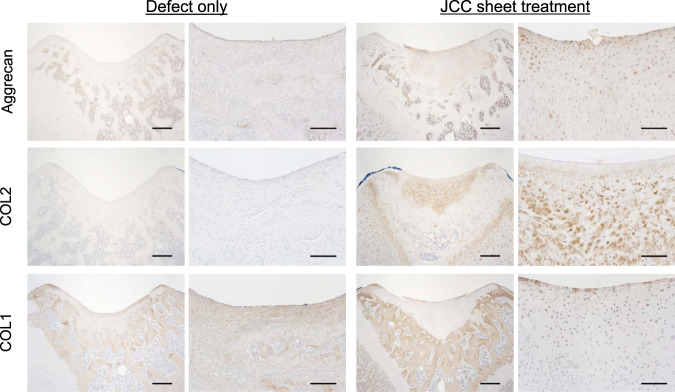
Cartilage-specific marker expression by transplanted human JCC sheet-derived tissue. Aggrecan staining (top), type II collagen staining (middle), type I collagen staining (bottom) are shown. All samples shown are 4 weeks after transplantation. Bars: Left columns of each treatment group: 500 µm, right columns of each treatment group: 100 µm.

**Fig. 6:**
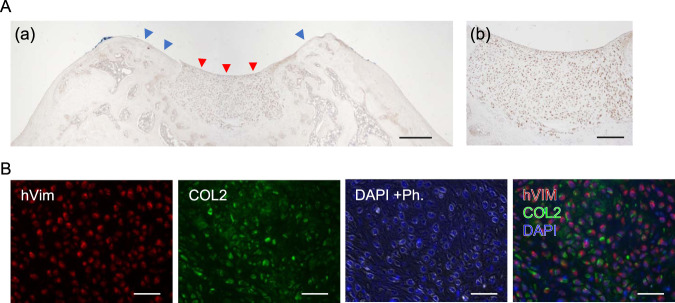
Transplanted human chondrocyte engraftment accompanied by type II collagen deposition. **A** (a) Human antigen-specific vimentin staining of a JCC sheet-treated sample. Red arrowheads indicate regenerated cartilage. Blue arrowheads indicate host cartilage. Bar: 500 µm (b) Magnified area of regenerated cartilage with human antigen-specific vimentin staining. Bar: 200 µm. **B** Double staining of human vimentin (red) and type II collagen (green). DAPI + Ph: DAPI and phase-contrast image to show nuclei (blue with white edge). The right panel shows the merged image of human vimentin, type II collagen, and DAPI. Bars: 200 µm. A histology sample of 4 weeks after treatment is shown.

The presence of aggrecan (ACAN) and type 2 collagen (COL2), hyaline cartilage-specific matrix proteins, and type 1 collagen (COL1), a damaged or arthritic articular cartilage marker was assessed on harvested knee samples by immunohistochemistry (IHC). The pannus at the defect site in the defect-only group showed strong COL1 expression, but ACAN and COL2 were not expressed (Fig. 5A). Regenerated neocartilage in the JCC sheet treatment group showed a limited expression of COL1 localized to neocartilage surfaces and abundant ACAN and COL2 expressions in the neocartilage body (Fig. 5B). We also used human-specific vimentin staining to determine the origin of the regenerated tissue. Specificity of human-specific anti-vimentin antibody was confirmed on superficial cartilage of fibrotic tissue in the nontreatment group by comparison to a “pan” vimentin antibody that cross-reacts to both human and rat cartilage (Supplementary Fig. 5). Human-vimentin-specific antibody did not react with rat cartilage or stromal tissue, whereas it reacted with the neocartilage tissue areas on JCC sheet treatment samples (Fig. 6A). Interestingly, COL2 was observed at the periphery of human-vimentin positive cells and adjacent interstitial matrix (Fig. 6B). These data strongly suggest that transplanted human JCC sheets are responsible for deposition of new cartilage matrix proteins and neocartilage generation.

**Fig. 7:**
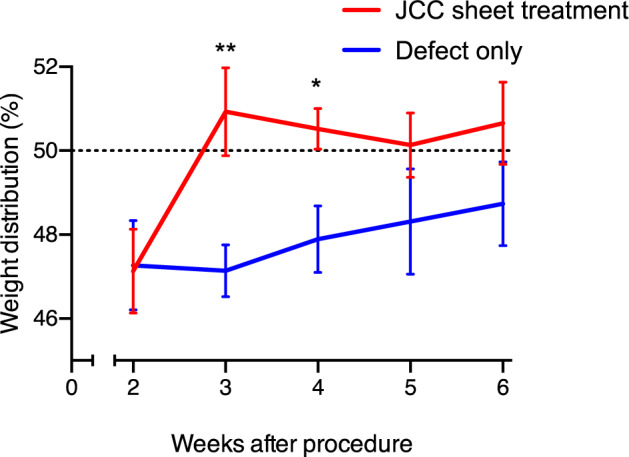
Rodent weight-bearing test. Weight distribution of rat left and right hind legs are measured, and percentages of treatment side weight bearing are shown. Sample numbers at week 2, 3, 4, 5, and 6 = 13, 12, 12, 8, and 8 (defect only group); = 16, 15, 15, 9, and 9 (defect + CS group), respectively. Data shown as mean and SEM. **p < 0.01, *p < 0.05 by Welch’s *t* test.

We assessed functional recovery induced by JCC sheet transplantation on the rat focal osteochondral defect models by measuring rat hind limb weight bearing. The JCC sheet treatment group showed rapid recovery in weight distribution on each leg after 3 weeks, while reduced weight bearing on the injured leg persisted in the defect-only group for over 6 weeks (Fig. 7 and Supplementary Fig. 6A), indicating that regenerated neocartilage ameliorates pain caused by the focal defect. JCC sheet transplantation did not affect total body weight (Supplementary Fig. 6B).

## Discussion

The many current clinical demands for treating cartilage defects are not satisfied by current tissue engineering products due to high graft costs, tissue availability, and poor adhesion techniques^31,32^. Osteochondral autograft transplantation is limited by (1) patient chondral integrity in the notch and peritrochlear region, (2) graft availability in large osteochondral defects, and has well-documented donor site morbidity. Autologous chondrocyte implantation (ACI) also sacrifices native patient cartilage and has significant preparation costs that proportionally increase with increasing patient treatment numbers. Moreover, ACI requires a two-stage surgical treatment for tissue harvest and subsequent processing/handling and re-implantation thereby resulting in delayed patient recovery and increased treatment costs. In contrast, fresh bulk osteochondral allograft transplantation (OCA) from deceased donors allows a single-stage treatment but is limited by chondral viability during storage and biological graft quality control, resulting in difficulty in uniform product preparation and limited tissue availability that may result in patient waiting months to years to obtain treatment. In contrast, JCCs can be obtained from surgical discards of juvenile donors without donor site morbidity. Moreover, cultured JCCs show high growth potential (Fig. 1D), low HLA levels (Fig. 3B), and can be cryopreserved as a cell bank, enabling the manufacturing of off-the-shelf JCC sheets that allow a single-stage chondral repair option that is not limited by graft size, availability, or defect location architecture. Finally, JCC sheets can be carefully controlled for product quality surveillance before patient treatment thereby ensuring little to no graft variability.

Clinical data reporting outcomes of ACI at 8–60 months observed that only 15% of the cohort shows type II collagen-dominant cartilage^33^. This low hyaline cartilage level may be attributed to the variable potency of transplanted autologous cells from individual adult patients and/or the cell delivery method using processed cell suspensions covered in situ by periosteal flaps or collagen membranes that may cause low cell density and variable defect retention. Autologous cultured chondrocytes on porcine collagen membrane (MACI) employs chondrocytes from patients’ own native cartilage in collagen I/III matrices for transplant into the chondral lesion. MACI has had promising clinical outcomes, but the superiority of MACI over other techniques remains unclear^34^. Six-month postoperative histological analysis shows hyaline-like cartilage rather than hyaline cartilage^35^. Chondrogenic differentiation is enhanced by cell condensation^36–38^. The hyaline-like regeneration in MACI may be attributable to the adult patients’ own cell origin and lack of cell–cell communication in the collagen matrix.

Chondrocytes are well-known to de-differentiate in passage cultures^39^. However, in contrast to adult chondrocytes, JCCs maintain higher proliferative capacity and differentiation potential after subculture^14,40^. Our results demonstrated that passaged JCCs exhibit an elongated cell morphology (Fig. 1C), but successfully redifferentiate to hyaline cartilage both in vitro (Figs. 1E and 2E) and in vivo (Fig. 4A, B). Interestingly, isolated JCCs also show stable cell growth over passage 10 (Figs. 1D and 3C). Collectively, these results, together with allogeneic cell sheet animal studies and many allograft studies in the knee, allow allogeneic transplantation of JCCs for cartilage regeneration, probably due to the fact of scarce accessibility of antigen-presenting cells and cytotoxic T lymphocytes to the regenerated cartilage tissue. Neocartilage forming capability and safety of highly passaged JCCs must be demonstrated in future preclinical studies and clinical focal cartilage defect treatments.

A single juvenile polydactyly donor can theoretically produce thousands of JCC cell sheets at P2 (Fig. 2F). In addition to the advantage of abundant cell sourcing for allogeneic transplantation^41,42^, we exploit the engineered JCC sheet production using commercial temperature-responsive cultureware that enables scalable, reliable harvesting and handling of intact allogeneic cell sheets and direct delivery of the tissue-like structure onto target tissue sites. Such scaffold-free tissue engineering approaches can avoid unexpected reaction/rejection of scaffold biomaterials and/or their degradation products^43,44^, which should enhance cartilage regeneration and integration in host tissue. Furthermore, JCC sheets are flexible and adjustable to fit on and into defect areas regardless of surface asperity, which can better facilitate graft integration with host tissue, a major issue in conventional osteochondral graft implantation, pellet/aggregate culture techniques, or biomaterial-based osteochondral repair^45^. Highly proliferative JCCs in the sheet occupy the defect area, show rapid and mature cartilage formation specifically at the defect area, and spontaneously close the gap with the host lateral interface of native cartilage in 4 weeks (Fig. 4A, B). Neocartilage formed by JCC sheets was substantially thicker than native rat cartilage (Fig. 4B), suggesting the innate developmental potential of human chondrocytes and their possible capacity to treat full-thickness chondral defects in human patients. Notably, the newly formed cartilage tissue did not display tumorigenic growth over 6 months (Fig. 4A, B), supporting the safety and stability of JCC sheets in situ. Chondrogenesis in embryonic development is a complex process initiated by MSC condensation on the bone-forming site, followed by terminal differentiation to mature cartilage^46^. In addition, contraction of MSC sheet promotes chondrogenic differentiation in vitro^38^. The mechanism of the differentiation process of transplanted JCC sheets may fundamentally follow this differentiation path, but future study must probe the detailed molecular interactions that compel neocartilage formation in situ.

Allogenic osteochondral grafts have been applied to many patients^47,48^ and previous allogeneic adult chondrocyte sheet transplantation models show cell retention in rabbits at 12 weeks^49^ and pigs at 3 months^50^. Allograft and allogeneic cell rejection are regulated by interactions between the donor HLA molecules and recipient T cells. Although class I MHC was expressed in JCC sheet cells (Fig. 3B), leukocyte number in synovial fluid is very low (<200 cells/μL in healthy and <5000 in osteoarthritis)^51^. Thus, the knee joint space is often considered an immune-privileged site. The graft failure in SD rats and long-term engraftment in nude rats suggests a critical role of T cells in a xenogeneic reaction to the implanted chondrocyte sheets in the immunocompetent rat knee. Possible mechanism in the observed xenogeneic rejection may be macrophage activation by T cells that recognize xenoantigens as previously described^52,53^. MHC II plays an important role in the immune rejection of transplanted allogeneic MSC via allo-antibody formation demonstrated with a class II transactivator knock-down model^54^; therefore, the absence of MHC II expression in the JCC sheet (Fig. 3B) suggests negligible chronic rejection and possible long-term engraftment in an allogeneic setting. In addition, cultured JCCs are reported to inhibit T cell proliferation and express inhibitory cell surface B7 co-stimulatory molecules^13^. Therefore, engineered JCC sheets might also be able to promote long-term cartilage regeneration with low immunogenicity as an allogeneic cell therapy.

In summary, we report that JCCs isolated from juvenile donor surgical discards can yield thousands of P2 JCC sheets, and we have confirmed consistent cell viability and quality, total cell numbers, surface markers, and neocartilage regeneration in vivo in a rodent focal osteochondral defect model. Beyond reliable cartilage formation, the method has scaling features amenable to allogeneic off-the-shelf production strategies, including intrinsically low HLA human cell sources, use of mass-handled commercial temperature-responsive cultureware, cell stability, expansion, banking and storage, and sheet durability in shipment or on-site fabrication adoption. These data strongly support the further applicability of JCC sheets in possibly addressing current substantial clinical unmet needs in focal chondral defect patients. Recent studies have revealed the molecular traits of human JCC sheets correlated with in vivo efficacy in rabbit osteochondral defect treatment^55^. Strategies that can validate regenerative capacity and minimize variability in new donor tissue sources without extensive in vitro and in vivo testing are needed before large-scale studies commence. In addition, better understanding the regenerative mechanisms of JCC sheets will allow more rational development of this reliable therapy and encourage future clinical adoption.

## Methods

### Cartilage sampling from juvenile human polydactyly donors

Cartilage from the phalanx and metacarpal bones of amputated polydactylous fingers and toes from 12 patients (24.8 ± 17.0 months old) (Table 1) was sharply dissected using a scalpel and maintained in saline immediately following harvest. All patients were prospectively enrolled at Intermountain Primary Children’s Hospital (Salt Lake City, USA). Institutional Review Board oversight from University of Utah and Intermountain Primary Children’s Hospital was waived due to use of deidentified routine surgical discards.

### Chondrocyte isolation

Cartilage harvested from juvenile donor tissues was transferred into saline, cut into <4 mm^2^ pieces by scalpel, and then incubated with 5 mg/mL of Type 1 collagenase at 37 °C for 1.5–3.0 h (LS004197, Worthington Biochemical, Lakewood, USA). Resulting cells were filtered through a 100-µm cell strainer, washed with saline and then resuspended in chondrocyte culture medium (DMEM-F12, 11320082, ThermoFisher Scientific, Waltham, USA) containing 1% antibiotic-antimycotic (15240062, ThermoFisher) and 20% fetal bovine serum (FBS) (16000044, ThermoFisher).

### Cell culture

Isolated chondrocytes were seeded on polystyrene dishes (CELLTREAT, Pepperell, USA) at 5000–10,000 cells/cm^2^ in a chondrocyte culture medium (described above). The medium was replaced with chondrocyte medium supplemented with 100 μg/mL L-ascorbic acid phosphate magnesium salt n-hydrate (013–19641, Fujifilm Wako Pure Chemical, Osaka, Japan) at the first medium change on day 4. Cells were passaged with this medium thereafter with the daily observation by phase-contrast microscopy throughout cell culture. Subconfluent cells were collected by TrypLE Select (12563011, ThermoFisher) dissociation and counted. Expanded cells were cryopreserved in STEM-CELLBANKER GMP grade (Zenoaq, Fukushima, Japan) at the end of P0. Serial subculture was performed with the thawed cells at the initial density of 10,000 cells/cm^2^ passaged every 3–5 days.

### Juvenile chondrocyte sheet preparation

Cell sheets were prepared from passage 1 cells sourced from thawed cryopreserved cells. Subconfluent P1 cells were collected with 1x TrypLE Select for 5 min, then seeded at a density of 10,000 cells/cm^2^ on temperature-responsive cell culture inserts (CellSeed, Tokyo, Japan). Chondrocyte culture media was changed every 3–4 days. After 2 weeks of culture, cell sheets were harvested with forceps after incubation at room temperature.

### Cell viability and total cell number of chondrocyte sheet

Single cells from fabricated JCC sheets were isolated by incubation with TrypLE Select for 15 min and 0.25 mg/mL collagenase P (11 213 857 001, Roche, Basel, Switzerland) for 30 min. Cells were counted via hemocytometer and cell viability was demonstrated via trypan blue (T8154, MilliporeSigma) ﻿dye exclusion^56^.

### Chondrogenic differentiation culture

Chondrogenic pellet culture was performed based on a previous study^38^. JCCs harvested at the end of P2 and P9 cultures or isolated cells from P2 JCC sheets were aliquoted in chondrocyte culture medium at 2.5 × 10^5^ cells in 15 mL conical tubes for pellet cultures. Tubes were spun at 500 × *g* for 10 min. Caps were loosened and cells were incubated at 37 °C, 5% CO_2_ for 3 days to facilitate pellet formation. After the 3-day incubation step, chondrogenic samples were induced with chondrogenic medium, control samples were replaced with a new chondrocyte culture medium, and all samples were transferred to a hypoxic incubator (37 °C, 5% CO_2_, 5% O_2_). Chondrogenic medium contained high glucose DMEM supplemented with 10 ng/mL transforming growth factor beta-3 (TGFβ3) (ThermoFisher), 200 ng/mL bone morphogenic protein-6 (BMP6) (PeproTech), 1% Insulin-Transferrin-Selenium (ITS-G) (ThermoFisher), 1% PS (Life Technologies), 1% non-essential amino acids (NEAA) (ThermoFisher), 100 nM dexamethasone (MP Biomedicals, Irvine, USA), 1.25 mg/ml bovine serum albumin (BSA) (MilliporeSigma), 50 μg/mL ﻿L-ascorbic acid phosphate magnesium salt n-hydrate﻿ (Fujifilm Wako Pure Chemical), 40 μg/mL L-proline (MilliporeSigma), and 5.35 μg/mL linoleic acid (MilliporeSigma). Media was changed twice a week for 3 weeks.

### In vitro cell transformation assay

In vitro cell transformation was evaluated by serial passage culture and soft agar assay. Juvenile chondrocytes were passaged up to 13 passages (57–67 days in total) with a seeding density of 10,000 cells/cm^2^. Cells were observed every day and cell growth rate was calculated as doubling time. Cell transformation was assessed by detecting anchorage-free proliferation. Cultured cells isolated from P2 chondrocyte sheets of normal culture periods (2 weeks) and extended culture periods (3.5 weeks) were seeded in a soft agar gel with chondrocyte culture media with ascorbate at a density of 5,000 cells per well by using CytoSelect Cell Transformation Assay (CBA-140, Cell Biolabs, San Diego, USA). Fluorescent signal representing cell number was measured with a spectrofluorometer (Cytation 3 image reader, BioTek, Winooski, USA) on day 0 and day 8 according to manufacturer’s protocol. HepG2 cells (HB-8065, ATCC) in DMEM containing 10% FBS and 1% antibiotic-antimycotic were used as positive control for anchorage-free cell growth. Data are shown as averages of relative fluorescent units from duplicate or triplicate assays.

### Flow cytometry

Isolated cell suspensions from chondrocyte sheets (dissociated as described before) were aliquoted and incubated in 1 µg/mL Fc block solution (564220, BD, Franklin Lakes, USA), resuspended in 10% FBS-containing PBS for 5–10 min, then labeled with fluorescent-conjugated antibodies (Supplementary Table 1) for 15 min with brief vortexing steps. Cells were washed with 10% FBS-containing PBS, centrifuged, resuspended with 1:1000 propidium iodide (PI) (556463, BD) in 10% FBS-containing PBS. Samples were analyzed with a cell analyzer (Canto, BD). Doublets were excluded with FSC-W and SSC-W gating, then the PI-negative population was analyzed for specific antibody staining. The gating strategy is shown in Supplementary Fig. 1.

### Histology

Fabricated cell sheets were fixed with 4% paraformaldehyde for 30 min at RT. Harvested rat knee tissue was fixed in 4% paraformaldehyde for four days and decalcified in RapidCal Immuno (BBC Biochemical, Mount Vernon, USA) for one day at RT. Samples were embedded in paraffin blocks and then cut into 5-µm transverse sections with a microtome. Slides were deparaffinized by baking in an oven at 65 °C and subsequent washes with xylene and ethanol. Sections were hydrated by gradual ethanol replacement by distilled water. Safranin-O was used for metachromatic staining for sulfated glycosaminoglycans. Samples were stained for 5 min with Wiegert’s Iron Hematoxylin (MilliporeSigma), 5 min with 0.5 g/L Fast Green FCF (MilliporeSigma), and 5 min with 0.1% Safranin-O (MilliporeSigma). Images were taken with a BX41 microscope (Olympus, Tokyo, Japan) and AmScope Software (USA).

### Immunohistochemistry

Sections of histology samples were hydrated, then antigen retrieval was performed. The retrieval method was chosen to preserve the tissue integrity of knee samples and cell sheet samples after the staining optimization: protease K (S3020, Agilent Technologies, Santa Clara, USA) for COL2 and vimentin staining of knee samples; heat antigen retrieval in citrate buffer (pH 6.0) (C9999, MilliporeSigma, Burlington, USA) for COL2 staining of cell sheet samples. Peroxidase blocking was performed with Hydrogen Peroxide Blocking Reagent (ab64218, Abcam, Cambridge, UK). After blocking with 5% donkey serum and 0.1% Triton-X in PBS for 1 h. Samples were then incubated overnight with primary antibodies at 4 °C. Polyclonal goat anti-type I collagen (1:200, SouthernBiotech, Birmingham, USA), monoclonal mouse anti-type II collagen (1:200, 2B1.5, ThermoFisher), polyclonal goat anti-aggrecan (1:100, AF1220, R&D Systems, Minneapolis, USA), and monoclonal rabbit anti-human vimentin (1:200, SP20, Abcam)^57,58^ were used as primary antibodies. Normal mouse IgG_2a_ (X0943, Agilent), normal goat IgG (NI02, MilliporeSigma), or normal rabbit IgG (X0903, Agilent) were used as isotype controls at the same concentration as the primary antibodies. Horseradish peroxidase (HRP)-conjugated goat anti-mouse antibody (1:1,000, 115–035–166, Jackson ImmunoResearch, West Grove, USA) was used for type II collagen. HRP-conjugated donkey anti-goat antibody (1:1,000, 705-035-147, Jackson) was used for type I collagen and aggrecan staining. HRP-conjugated goat anti-rabbit antibody (1:1,000, 111-035-144, Jackson) was used for human vimentin staining. ImmPACT DAB Peroxidase (HRP) Substrate (SK-4105, Vector Laboratories, Burlingame, USA) was used as a chromogen. Brightfield images were taken with a BX41 microscope and AmScope Software. Fluorescent images were taken with Axio Vert.A1 microscope and ZEN software (Zeiss, Oberkochen, Germany). All primary and secondary antibodies are listed in Supplementary Tables 2 and 3, respectively.

### Surgical and transplantation procedure

The animal study plan was evaluated and approved by Institutional Animal Care & Use Committee (IACUC, University of Utah) (assigned ID: 17-09011). SD rats and nude rats (6-week-old), male and female, were purchased from Charles River Laboratories (Wilmington, USA). After a week of acclimatization at the animal facility, animals were anesthetized using isoflurane and O_2_ gas. A medial parapatellar incision was made to expose the knee joint; the patella was laterally dislocated and a focal osteochondral defect (diameter 2 mm; depth 200–350 μm) was created on the patellar groove of the femur using an electric grinder without penetration to the bone marrow. Defect depth was controlled by the procedure under a surgical stereo zoom microscope (SZX10, Olympus, Japan) with repeated depth measurement with a needle tip (25 G, BD). Chondrocyte sheets prepared in temperature-responsive cell culture inserts in six-well plates (described above) were washed with saline, then cut in halves by a razor and single sheet halves transplanted to each surgical knee defect after defect creation. All animals received buprenorphine for 2 days and carprofen for 3 days in compliance with the IACUC protocol. Animals were sacrificed after 4, 8, 12, and 24 weeks for further histological evaluations.

### Rat hind limb weight-bearing distribution assessment

Weight distribution in rats was demonstrated with Incapacitance Tester (Linton Instrumentation, UK), a device with two separate boards on which the rats sit, measuring how the rats distribute their weight. All animals were acclimatized 1–2 times before and after surgery. No measurement was done until 2-week time point after surgery to avoid effects of muscle trauma and analgesia. Weight distribution was calculated by the following formula:

$$Weight\;distribution\left( \% \right) = \left( {Treated\;side\prime s\;load} \right)/(Treated\;side\prime s\;load + intact\;side\prime s\;load) \ast 100$$

Sample numbers at week 2, 3, 4, 5, and 6 = 13, 12, 12, 8, and 8 (nontreatment group); = 16, 15, 15, 9, and 9 (defect + CS group), respectively. Data are shown as mean and SEM. **p < 0.01, *p < 0.05 by Welch’s *t* test.

### Reporting Summary

Further information on research design is available in the Nature Research Reporting Summary linked to this article.

## Supplementary information

Supporting Information

Reporting Summary

## Data Availability

Data sets generated and/or analyzed during the current study are available from the corresponding author upon reasonable request.
