# Supplementary material for: Safety and efficacy of human juvenile chondrocyte-derived cell sheets for osteochondral defect treatment
Source: NPJ Regen Med. 2021 Oct 15;6:65. doi: 10.1038/s41536-021-00173-9 (PMC8520001; doi:10.1038/s41536-021-00173-9)

ADDITIONAL INFORMATION

Supplementary Figure 1. Gating strategy of flow cytometry analysis. First box shows total events. Second and third boxes show the doublet exclusion by eliminating high forward scatter width and side scatter width, respectively. Fourth box shows dead cell exclusion by propidium iodide (PI) staining. PI negative population was used for cell surface marker analysis in Fig 3B. At least 30,000 events were obtained for each target analysis.

Supplementary Figure 2. Histological analysis of P9 juvenile chondrocyte sheet. Hematoxylin and eosin staining, Safranin-O staining, Toluidine blue staining, aggrecan, type I collagen, and type II collagen immunohistochemistry of P9 JCC sheets are shown. Bars: 50 µm.

Supplementary Figure 3. Transplantation results of Sprague Dawley rats. (A) macroscopic images of patellofemoral groove defects with or without JCC sheet treatment. (B) Safranin O staining of cartilage samples at 4 weeks post operation. Rxx indicates animal ID.

Supplementary Figure 4. Safranin-O staining of native knee and defect-created knee. (A) and (D) Native knee tissue without any treatment. (B) and (E) Immediately after the defect creation. (C) and (F) Two days after the defect creation. Bars: 500 µm (A-C), 100 µm (D-F).

Supplementary Figure 5. Validation of human-specific vimentin antibody staining. Left column shows human vimentin antigen-specific staining. Right column shows species cross-reacting vimentin staining. Top row: No defect native rat cartilage. Middle row:

JCC sheet treatment group harvested at 4 weeks post operation. Bottom row: defect only group at 4 weeks post operation.

Supplementary Figure 6. All measured values of rat weight bearing experiment. (A) All raw data of calculated weight distributions of 2-4 weeks after the treatment. X-axis shows weight distribution (%) on the treated hind leg. Y-axis shows animal ID. Circles: JCC sheet treatment group (top); Triangles: Defect only group (bottom). (B) Fold change of body weight after treatment. Blue: defect only group, Red: JCC sheet treatment group.

Supplementary Table 1. Antibodies used in flow cytometry

Targets	Conjugate	Vendor	Cat #	Clone	Lot	Concentration	Dilution factors
CD45	PB	Biolegend	304021	HI30 (IgG1κ)	B225702	500 µg/mL	5:100
HLA-ABC	PB	Biolegend	311417	W6/32 (IgG2a)	B191432	500 µg/mL	3:100
CD31	AF488	Biolegend	303109	WM59 (IgG1κ)	B190516	400 µg/mL	5:100
HLA -DR, -DP, -DQ	FITC	Biolegend	361705	Tu39 (IgG2a)	B228758	200 µg/mL	5:100
CD44	PE	Biolegend	338807	BJ18 (IgG1κ)	B222834	50 µg/mL	1:100
CD90	PE	Biolegend	328109	5E10 (IgG1κ)	B206721	50 µg/mL	1:100
CD106	APC	Biolegend	305809	STA (IgG1κ)	B208208	100 µg/mL	5:100
CD81	APC	BD	551112	JS-81 (IgG1κ)	8005529	12.5 µg/mL	20:100
Lineage (CD3, CD14, CD16, CD19, CD20, CD56)	APC	Biolegend	348803	Mix of 6 ab (CD3, CD14, CD16, CD19, CD56: IgG1κ, CD20: IgG2b)	B199913	IgG1: 99.25 µg/mL IgG2b 6.25 µg/mL	20:100
Normal IgG1κ	PB	Biolegend	400131	MOPC-21 (IgG1κ)	B229538	500 µg/mL	Set as same conc. as sample
Normal IgG2a	PB	Biolegend	400235	MOPC-173 (IgG2a)	B243657	500 µg/mL	Set as same conc. as sample
Normal IgG1κ	AF488	Biolegend	400129	MOPC-21 (IgG1κ)	B220820	200 µg/mL	Set as same conc. as sample
Normal IgG2a	FITC	Biolegend	400207	MOPC-173 (IgG2a)	B235551	500 µg/mL	Set as same conc. as sample
Normal IgG1κ	PE	Biolegend	400111	MOPC-21 (IgG1κ)	B244596	200 µg/mL	Set as same conc. as sample
Normal IgG1κ	APC	Biolegend	400120	MOPC-21 (IgG1κ)	B257952	200 µg/mL	Set as same conc. as sample
Normal IgG2b	APC	Biolegend	400319	MPC-11 (IgG2b)	B202284	200 µg/mL	Set as same conc. as sample

Supplementary Table 2. Primary antibodies used in immunohistochemistry

Target	Host	Clone/ID	Dilution factors	Original conc.	Vendor/ producer	Cat #
COL II	Mouse	2B1.5 (IgG2a)	1:200	200 µg/mL	Invitrogen (Thermo)	MA5-12789
COL I	Goat	poly	1:200	400 µg/mL	Southern Biotech	1310-01
Aggrecan	Goat	poly	1:100	200 µg/mL	R&D	AF1220
Human Vimentin	Rabbit	SP20	1:200	10 - 50 µg/mL	Abcam	ab16700
Vimentin	Rabbit	EPR3776	1:400	264 - 268 µg/ml	Abcam	Ab92547
Isotype	Mouse	IgG2a	1:100	100 µg/mL	Dako/Agilent	X0943
Isotype	Mouse	IgG1κ DAK-GO1	1:10	100 µg/mL	Dako/Agilent	X0931
Isotype	Goat	-	1:50	100 µg/mL	Chalbiochem /Merck	NI02-100UG
Isotype	Rabbit	-	1:200,000	15 mg/mL	Dako	X0936

Supplementary Table 3. Secondary antibodies used in immunohistochemistry

Target	Host	Conjugate	Dilution factors	Original conc.	Vendor	Cat #
Mouse IgG	Goat	HRP	1:1000	0.8 mg/mL	Jackson	115-035-166
Goat IgG	Donkey	HRP	1:1000	0.8 mg/mL	Jackson	705-035-147
Rabbit IgG	Goat	HRP	1:1000	0.8 mg/mL	Jackson	111-035-144
Mouse IgG	Donkey	AF488	1:500	2 mg/mL	Invitrogen (Thermo)	A-21202
Rabbit IgG	Goat	AF568	1:500	2 mg/mL	Invitrogen (Thermo)	A-11011

Supplementary Figure 1. Gating strategy of flow cytometry analysis

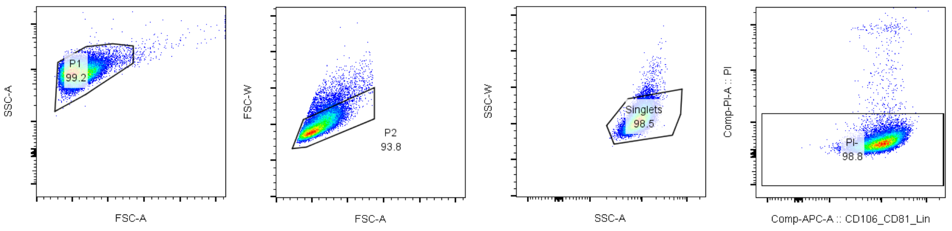

Supplementary Figure 2. Histological analysis of P9 juvenile chondrocyte sheet

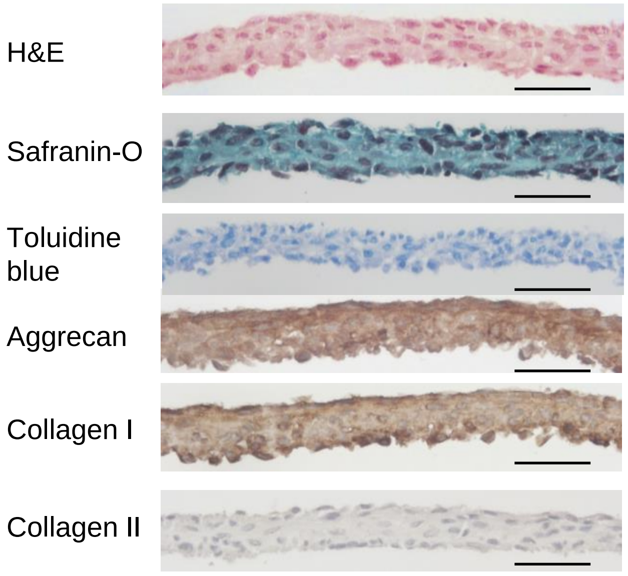

Supplementary Figure 3. Transplantation results of Sprague Dawley rats.

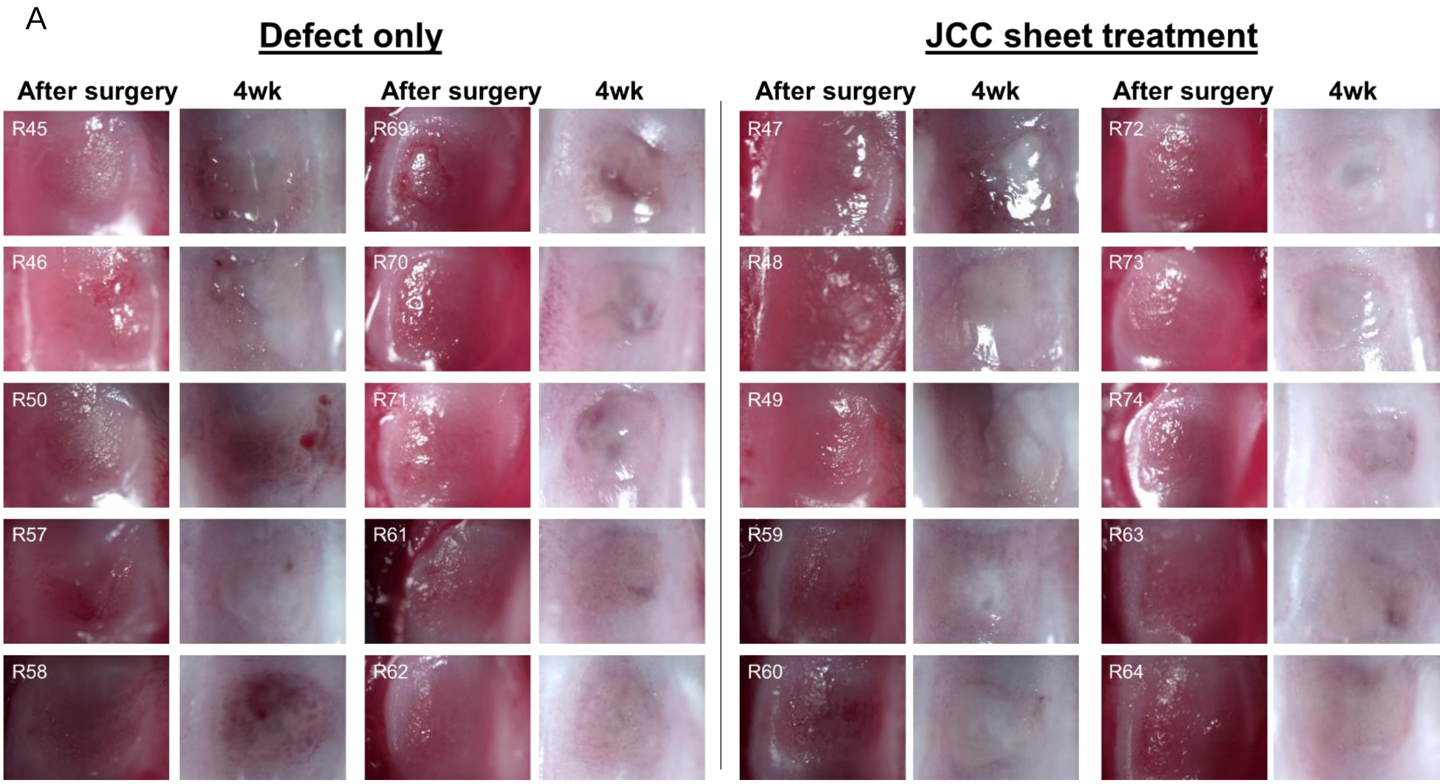

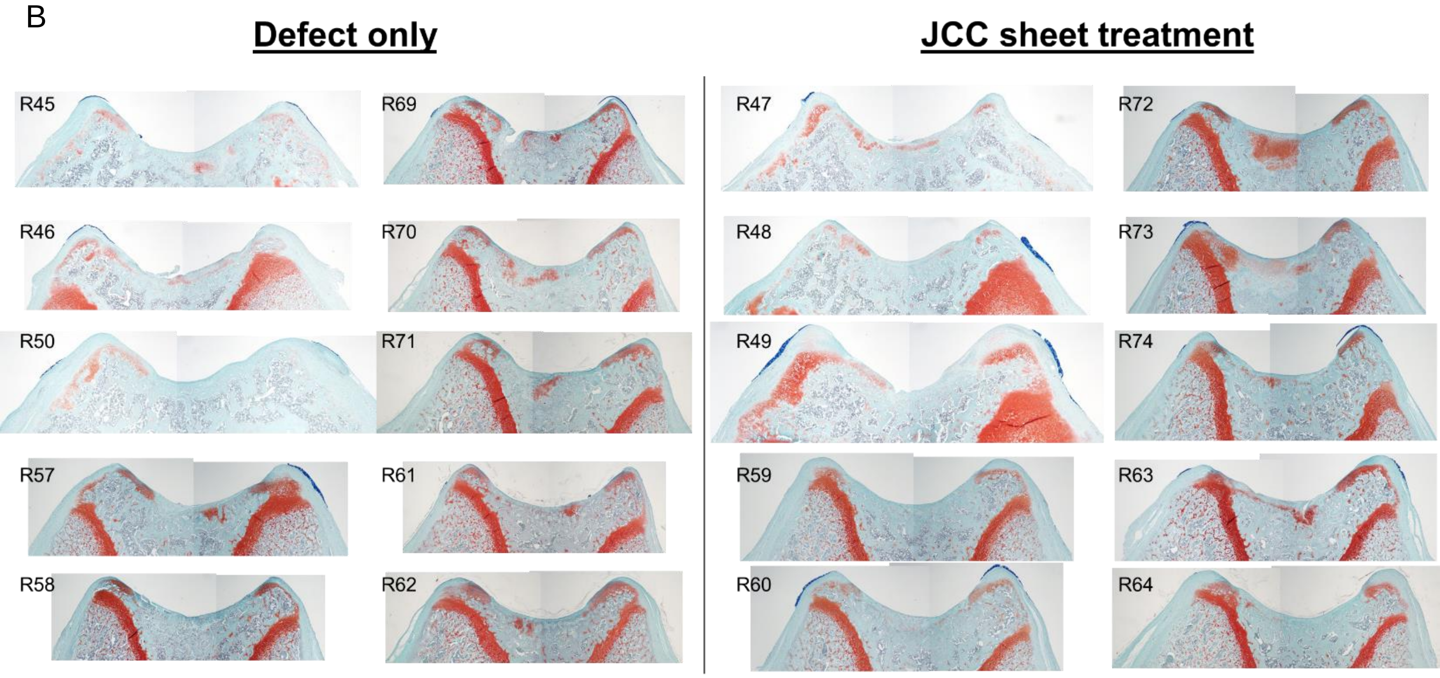

Supplementary Figure 4. Safranin-O staining of native knee and defect-created knee

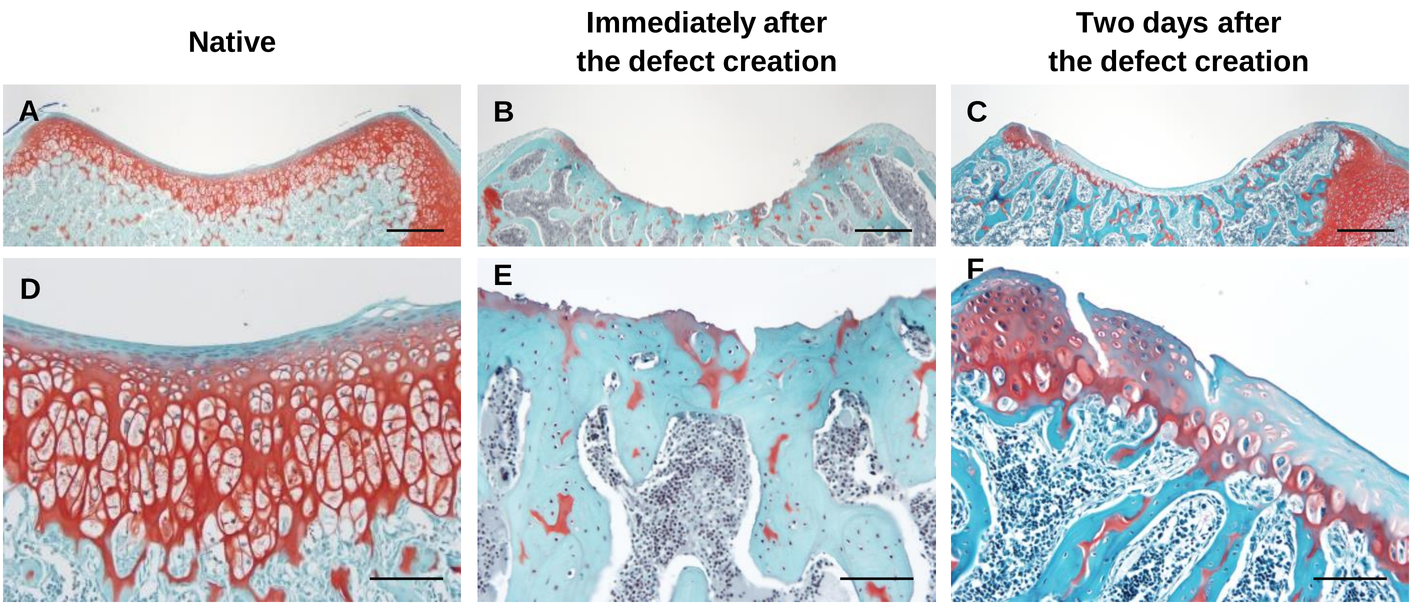

Supplementary Figure 5 . Validation of human specific vimentin antibody staining

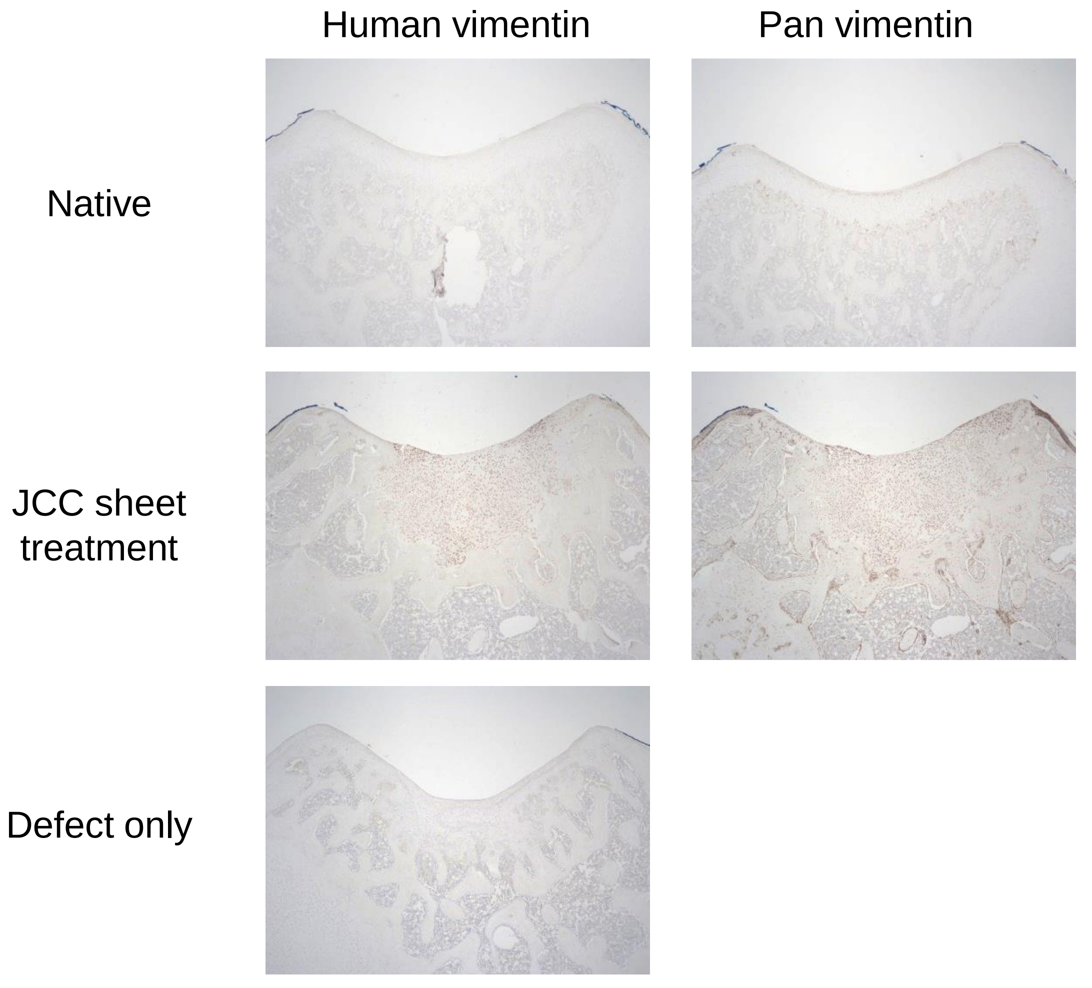

Supplementary Figure 6. All measured values of rat weight bearing experiment.

A

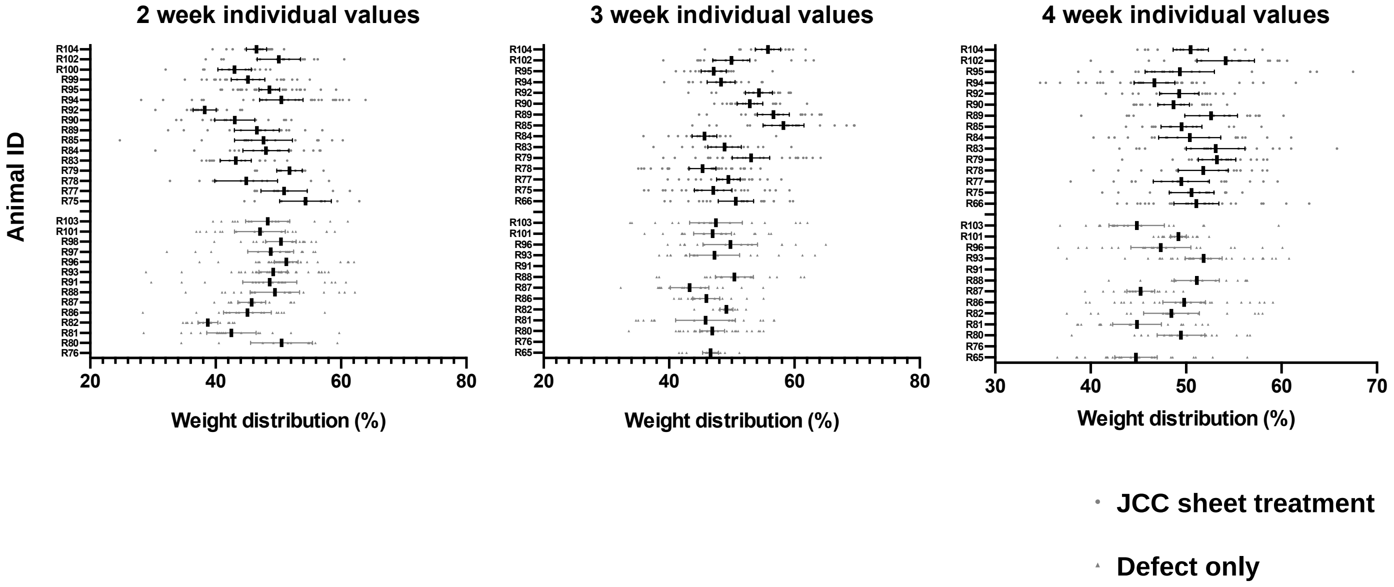

B

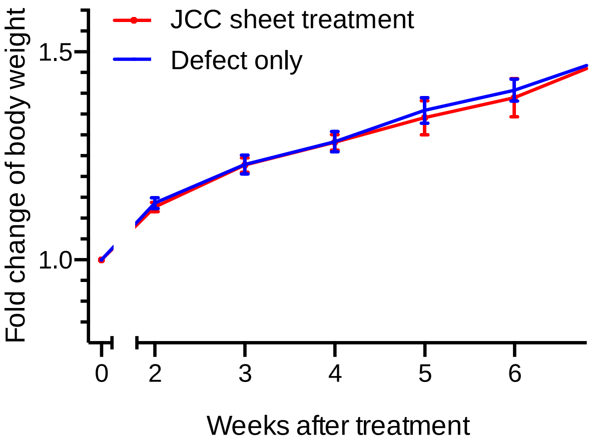

Supplement: Supplementary file 1 — Supporting Information [file 41536_2021_173_MOESM1_ESM.pdf]
