# Supplementary material for: Safety and efficacy of human juvenile chondrocyte-derived cell sheets for osteochondral defect treatment
Source: NPJ Regen Med. 2021 Oct 15;6:65. doi: 10.1038/s41536-021-00173-9 (PMC8520001; doi:10.1038/s41536-021-00173-9)
Supplement: Supplementary file 2 — Reporting Summary [file 41536_2021_173_MOESM2_ESM.pdf]

nature research

Corresponding author(s): Makoto Kondo

Last updated by author(s): Mar 26, 2021

Reporting Summary

Nature Research wishes to improve the reproducibility of the work that we publish. This form provides structure for consistency and transparency in reporting. For further information on Nature Research policies, see our Editorial Policies and the Editorial Policy Checklist.

Statistics

For all statistical analyses, confirm that the following items are present in the figure legend, table legend, main text, or Methods section.

n/a	Confirmed	
☐	☒	The exact sample size (*n*) for each experimental group/condition, given as a discrete number and unit of measurement
☐	☒	A statement on whether measurements were taken from distinct samples or whether the same sample was measured repeatedly
☐	☒	The statistical test(s) used AND whether they are one- or two-sided<br>*Only common tests should be described solely by name; describe more complex techniques in the Methods section.*
☐	☒	A description of all covariates tested
☒	☐	A description of any assumptions or corrections, such as tests of normality and adjustment for multiple comparisons
☐	☒	A full description of the statistical parameters including central tendency (e.g. means) or other basic estimates (e.g. regression coefficient) AND variation (e.g. standard deviation) or associated estimates of uncertainty (e.g. confidence intervals)
☐	☒	For null hypothesis testing, the test statistic (e.g. *F*, *t*, *r*) with confidence intervals, effect sizes, degrees of freedom and *P* value noted<br>*Give P values as exact values whenever suitable.*
☒	☐	For Bayesian analysis, information on the choice of priors and Markov chain Monte Carlo settings
☒	☐	For hierarchical and complex designs, identification of the appropriate level for tests and full reporting of outcomes
☒	☐	Estimates of effect sizes (e.g. Cohen's *d*, Pearson's *r*), indicating how they were calculated

Our web collection on statistics for biologists contains articles on many of the points above.

Software and code

Policy information about availability of computer code

Data collection	Flow cytometry samples were analyzed with a cell analyzer (Canto, BD).<br>Fluorescent signal representing cell number was measured with a spectrofluorometer (Cytation 3 image reader, BioTek)<br>Weight distribution in rats was demonstrated with Incapacitance Tester, Linton Instrumentation.
Data analysis	Numeric data was summarized with Microsoft Excel or GraphPad Prism 7.<br>Software used for statistical analysis was GraphPad Prism 7.<br>FACS analysis was performed with FlowJo v10 software.

For manuscripts utilizing custom algorithms or software that are central to the research but not yet described in published literature, software must be made available to editors and reviewers. We strongly encourage code deposition in a community repository (e.g. GitHub). See the Nature Research guidelines for submitting code & software for further information.

Data

Policy information about availability of data

All manuscripts must include a data availability statement. This statement should provide the following information, where applicable:
- Accession codes, unique identifiers, or web links for publicly available datasets
- A list of figures that have associated raw data
- A description of any restrictions on data availability

Data sets generated and/or analyzed during the current study are available from the corresponding author upon reasonable request.

Field-specific reporting

Please select the one below that is the best fit for your research. If you are not sure, read the appropriate sections before making your selection.

☒ Life sciences ☐ Behavioural & social sciences ☐ Ecological, evolutionary & environmental sciences

For a reference copy of the document with all sections, see nature.com/documents/nr-reporting-summary-flat.pdf

Life sciences study design

All studies must disclose on these points even when the disclosure is negative.

Sample size	We use the minimum number of animals or cell samples necessary to fulfill statistical significance. We determined required sample sizes using preliminary data of defect model that showed no regeneration in any of the samples.
Data exclusions	All technically validated data was used.
Replication	All of our findings were technically and biologically replicated and reproduced multiple times.
Randomization	Animals were allocated randomly into the different experimental group.
Blinding	Treatment condition was blinded during the analyses of histology and weight bearing.

Reporting for specific materials, systems and methods

We require information from authors about some types of materials, experimental systems and methods used in many studies. Here, indicate whether each material, system or method listed is relevant to your study. If you are not sure if a list item applies to your research, read the appropriate section before selecting a response.

Materials & experimental systems

n/a	Involved in the study	
☐	☒	Antibodies
☐	☒	Eukaryotic cell lines
☒	☐	Palaeontology and archaeology
☐	☒	Animals and other organisms
☒	☐	Human research participants
☒	☐	Clinical data
☒	☐	Dual use research of concern

Methods

n/a	Involved in the study	
☒	☐	ChIP-seq
☐	☒	Flow cytometry
☒	☐	MRI-based neuroimaging

Antibodies

Antibodies used

FLOW CYTOMETRY
CD45 PB Biolegend 304021 HI30 (IgG1κ) B225702
HLA-ABC PB Biolegend 311417 W6/32 (IgG2a) B191432
CD31 AF488 Biolegend 303109 WM59 (IgG1κ) B190516
HLA -DR, -DP, -DQ FITC Biolegend 361705 Tu39 (IgG2a) B228758
CD44 PE Biolegend 338807 BJ18 (IgG1κ) B222834
CD90 PE Biolegend 328109 5E10 (IgG1κ) B206721
CD106 APC Biolegend 305809 STA (IgG1κ) B208208
CD81 APC BD 551112 JS-81 (IgG1κ) 8005529
Lineage (CD3, CD14, CD16, CD19, CD20, CD56) APC Biolegend 348803 Mix of 6 ab
(CD3, CD14, CD16, CD19, CD56: IgG1κ, CD20: IgG2b) B199913
Normal IgG1κ PB Biolegend 400131 MOPC-21 (IgG1κ) B229538
Normal IgG2a PB Biolegend 400235 MOPC-173 (IgG2a) B243657
Normal IgG1κ AF488 Biolegend 400129 MOPC-21 (IgG1κ) B220820
Normal IgG2a FITC Biolegend 400207 MOPC-173 (IgG2a) B235551
Normal IgG1κ PE Biolegend 400111 MOPC-21 (IgG1κ) B244596
Normal IgG1κ APC Biolegend 400120 MOPC-21 (IgG1κ) B257952
Normal IgG2b APC Biolegend 400319 MPC-11 (IgG2b) B202284

IMMUNOHISTOCHEMISTRY
COL II Mouse 2B1.5 (IgG2a) 1:200, 200 μg/mL Invitrogen (Thermo) MA5-12789
COL I Goat poly 1:200, 400 μg/mL Southern Biotech 1310-01
Aggrecan Goat poly 1:100, 200 μg/mL R&D AF1220
Human Vimentin Rabbit SP20 1:200, 10 - 50 μg/mL Abcam ab16700

April 2020

Vimentin Rabbit EPR3776 1:400, 264 - 268 µg/ml Abcam Ab92547
Isotype Mouse IgG2a 1:100, 100 µg/mL Dako/Agilent X0943
Isotype Mouse IgG1κ DAK-GO1 1:10, 100 µg/mL Dako/Agilent X0931
Isotype Goat - 1:50, 100 µg/mL Chalbiochem/Merck NI02-100UG
Isotype Rabbit - 1:200000, 15 mg/mL Dako X0936

SECONDARY ANDIBODIES
Mouse IgG Goat HRP 1:1000, 0.8 mg/mL Jackson 115-035-166
Goat IgG Donkey HRP 1:1000, 0.8 mg/mL Jackson 705-035-147
Rabbit IgG Goat HRP 1:1000, 0.8 mg/mL Jackson 111-035-144
Mouse IgG Donkey AF488 1:500 2 mg/mL Invitrogen (Thermo) A-21202
Rabbit IgG Goat AF568 1:500 2 mg/mL Invitrogen (Thermo) A-11011

Validation

Antibodies were validated according to manufacture's description. Specific validation of human vimentin antibody is shown in Supplemental Figure 5.

Eukaryotic cell lines

Policy information about cell lines

Cell line source(s)	HepG2
Authentication	This cell line was purchased from ATCC and used within minimal passage.
Mycoplasma contamination	The cell line was not tested for mycoplasma contamination.
Commonly misidentified lines (See ICLAC register)	We used this cell line in in vitro transformation assay as a anchorage-independent growing cell control. HepG2 is reidentified as hepatoblastoma cell line instead of hepatocyte carcinoma cell line (Lopez-Terrada et al 2009, PMID 19751877). Either case fulfills the purpose of use in our study.

Animals and other organisms

Policy information about studies involving animals; ARRIVE guidelines recommended for reporting animal research

Laboratory animals	Sprague Dawley rats and nude (RNU) rats (6-week old) male and female, were purchased from Charles River Laboratories (Wilmington, USA). After a week of acclimatization at the animal facility, animals were employed for knee osteochondral defect regeneration study.
Wild animals	This study did not involve wild animals.
Field-collected samples	This study did not involve samples collected from the field.
Ethics oversight	The animal study plan was evaluated and approved by Institutional Animal Care & Use Committee (IACUC, University of Utah) (assigned ID: 17-09011).

Note that full information on the approval of the study protocol must also be provided in the manuscript.

Flow Cytometry

Plots

Confirm that:

☒ The axis labels state the marker and fluorochrome used (e.g. CD4-FITC).

☒ The axis scales are clearly visible. Include numbers along axes only for bottom left plot of group (a 'group' is an analysis of identical markers).

☒ All plots are contour plots with outliers or pseudocolor plots.

☒ A numerical value for number of cells or percentage (with statistics) is provided.

Methodology

Sample preparation	Single cells from fabricated JCC sheets were isolated by incubation with TrypLE Select for 15 min and 0.25 mg/mL collagenase P (11 213 857 001, Roche, Basel, Switzerland) for 30 min. Cells were counted via hemocytometer and cell viability was demonstrated via trypan blue (T8154, MilliporeSigma) dye exclusion before allocating flow cytometry analysis.
Instrument	Flow cytometry samples were analyzed with a cell analyzer (Canto, BD).
Software	FACS analysis was performed with FlowJo v10 software.
Cell population abundance	All analysis was performed on the isolated cells from cultured cell sheets prepared at passage 2. More than 30,000 events were acquired. Vast majority of the events were located in the gate of PI-negative population.

Gating strategy	Doublets were excluded with FSC-W and SSC-W gating, then the PI-negative population was analyzed. Positive/negative threashold was determined by isotype control samples with the same number event acquisition. Isotype control histograms are overlaid on the sample data in the figure. Gating strategy is shown in Supplemental Figure 1.

☒ Tick this box to confirm that a figure exemplifying the gating strategy is provided in the Supplementary Information.
